# Multiple-Site Hemodynamic Analysis of Doppler Ultrasound with an Adaptive Color Relation Classifier for Arteriovenous Access Occlusion Evaluation

**DOI:** 10.1155/2014/203148

**Published:** 2014-04-30

**Authors:** Jian-Xing Wu, Yi-Chun Du, Ming-Jui Wu, Chien-Ming Li, Chia-Hung Lin, Tainsong Chen

**Affiliations:** ^1^Department of Biomedical Engineering, National Cheng Kung University, Tainan City 70101, Taiwan; ^2^Department of Electrical Engineering, Southern Taiwan University of Science and Technology, Tainan City 71005, Taiwan; ^3^Department of Internal Medicine, Kaohsiung Veterans General Hospital, Tainan Branch, Tainan City 71051, Taiwan; ^4^Division of Infectious Diseases, Department of Medicine of Chi Mei Medical Center, Tainan City 71004, Taiwan; ^5^Department of Electrical Engineering, Kao Yuan University, Kaohsiung City 82151, Taiwan

## Abstract

This study proposes multiple-site hemodynamic analysis of Doppler ultrasound with an adaptive color relation classifier for arteriovenous access occlusion evaluation in routine examinations. The hemodynamic analysis is used to express the properties of blood flow through a vital access or a tube, using dimensionless numbers. An acoustic measurement is carried out to detect the peak-systolic and peak-diastolic velocities of blood flow from the arterial anastomosis sites (A) to the venous anastomosis sites (V). The ratio of the supracritical Reynolds (Re_supra_) number and the resistive (Res) index quantitates the degrees of stenosis (DOS) at multiple measurement sites. Then, an adaptive color relation classifier is designed as a nonlinear estimate model to survey the occlusion level in monthly examinations. For 30 long-term follow-up patients, the experimental results show the proposed screening model efficiently evaluates access occlusion.

## 1. Introduction

Chronic renal failure is an irreversible and progressive disease. In Taiwan, more than 70 thousand people need to receive hemodialysis treatment and this number is increasing year by year. Arteriovenous access, such as Brescia-Cimino arteriovenous fistulas (AVFs) or polytetrafluoroethylene grafts (AVGs), is a vital access for hemodialysis therapy. Maintenance of proper function of intragraft blood flow (IBF), <600 mL/min for AVF and <400–500 mL/min for AVG, is the most important issue for end-stage renal disease patients. Due to repeated puncturing of this access every two days or long-term use, access occlusion and failures are caused by inadequate arterial inflow or venous outflow occlusion. This causes thrombosis, resulting in intimal hyperplasia, chronic fibrin, cellular deposits, and aneurysm [1, 2]. Narrowing of the interior of the access increases the stress on the vascular wall and produces vibration, turbulent flow, and high-pressure pulsatile flow.

For early detection and homecare applications, previous studies have shown that the occlusion produces audible vascular murmurs, resulting in increased new high-frequency components in the power spectra [3–7]. Stethoscope auscultation provides a noninvasive diagnostic technique to detect high-pitch murmurs with existing access occlusion. However, vascular murmurs are regarded as random and stochastic signals and are difficult to detect in the time domain. Frequency domain and time-frequency techniques, such as the maximum entropy method (MEM) [4, 5], Burg autoregressive method [6, 7], and Fourier transform (FT) wavelet transform (WT) [6–10], are used to extract the specific frequencies and the magnitude of the characteristic frequencies. Then, the decision-making diagnosis systems with artificial neural networks, support vector machines, and fuzzy petri nets are carried out to perform the classification tasks. However, phonography techniques are affected by the measurement sites, the sizes of the sampling window, and the parametric mathematical models for phonoangiography (PCG) signal analysis. In addition, Doppler sonography, intravascular ultrasound image, colour duplex ultrasound, and X-ray angiography [11–13] are also used to detect the occlusion in these vascular accesses in clinical examinations. Stationary instruments provide reliable techniques and high accuracy in clinical environments. However, they need to appreciate the operational principles and require extra learning due to the limitations of the patients themselves.

The ultrasound flowmeter is also a reliable and highly accurate technique and provides a good resolution in detecting blood properties [14–16]. However, the Doppler power and velocity across an access under a pulsatile blood flow and the ultrasonic backscattering signal from pulsatile flowing blood are affected by the shear rate, as well as the acceleration of the flow velocity and the turbulences. To improve ultrasonic signal quality and the SNR at a specific depth, Doppler ultrasound with a bipolar pulse drive can achieve the best resolutions in high- and low-frequency ranges of 20 MHz to 50 MHz and 8 MHz to 20 MHz, respectively, which is enough to characterize the properties of the blood flow through surface and deep vessels. It is well developed, compact, fast, and free of irradiation [15–17]. The purpose of this study is to use the Doppler ultrasound device for measuring blood flow, such as peak-systolic velocity, peak-diastolic velocity, end-diastolic velocity, and heart rate. Then the dimensionless numbers, supracritical Reynolds (*Re*_supra_) number and resistive index (Res), are computed using flow velocities and the quantitative degrees are applied to screen the DOS. Color relational analysis (CRA) [18], as a classifier, performs the recognition task for assessment of arteriovenous access occlusion, using a hue-saturation-value color model [19]. It has a flexible pattern mechanism with add-in and delete-off training data and does not demand strict statistical methods and inference rules in real time screening applications [18]. An adaptive modeling system with an adjusting recognition coefficient to enhance the patency rate using a particle swarm optimization (PSO) algorithm was utilized to increase the accuracy [20, 21]. For 30 follow-up patients, the results show the proposed screening system is more efficient for occlusion evaluation.

The rest of this paper is organized as follows: Section 2 addresses the measurement technological support and experimental setup, and Section 3 describes the adaptive color relation classifier. In Sections 4 and 5, experimental results and conclusion are provided and the efficiency of the proposed screening method is demonstrated.

## 2. Measurement Technological Support and Experimental Setup

### 2.1. Doppler Ultrasound Measurement

**Figure 1:**
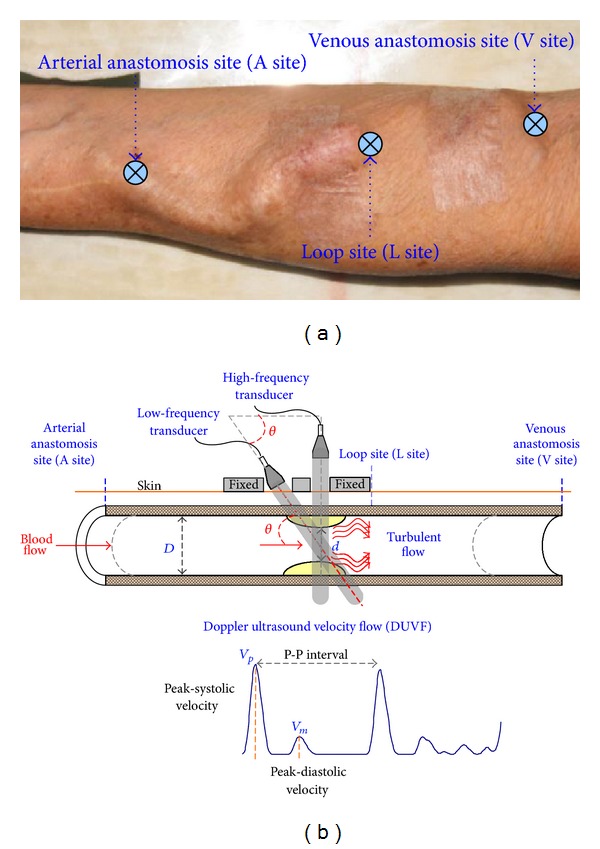
(a) Arteriovenous shunt and its measurement sites, (b) Doppler ultrasound measurements for ultrasound images and flow velocities.

In clinical examinations, auscultation and ultrasound examinations are used to detect the arteriovenous shunt (AVS) occlusion. Ultrasound examinations, such as Doppler ultrasound imaging, intravascular ultrasound imaging, and ultrasound flowmeter [22, 23], are reliable and highly accurate clinical techniques. It can operate in A-scan and B-scan instrument, using low-frequency and high-frequency sound waves, which can be processed to create quantitative images for marking the vessel axis and diameter of the 2D ultrasound image [24, 25], and also provides a good resolution in detecting blood properties, such as flow velocities, aggregation, and coagulation [14–16], as shown in Figure 1. In a follow-up examination, it can also be used to determine whether an abnormality is stable or a treatment is working. Among these, the ultrasound flowmeter is noninvasive, well developed, compact, and free of irradiation measurement technique. The purpose of this study is to use the Doppler ultrasound device for measurements of physiologic blood flow in AVS access, such as Doppler ultrasound velocity flow, peak-systolic velocity, peak-diastolic velocity, end-diastolic velocity, and heart rate.

Figure 1 shows the Doppler angles correct with flow velocities that can be estimated from the Doppler shift. The maximum frequency, *f*_max⁡_, and mode frequency of Doppler spectra are less changed along with the change in the sample volume length when setting the sample volume position at the center of the blood vessel. The flow velocity within the sample volume is measured as [26]

(1) $$\begin{array}{c} V=\frac{f_{d}c}{2f_{0}\cos\theta }, \end{array}$$

where *c* is the speed of sound in the blood, the nominal velocity of ultrasound in tissue is approximately 1540 m/sec, *f*_*d*_ is the Doppler shift frequency, *f*_0_ is the center frequency of transmitted ultrasound, and *θ* is the Doppler angle, 50~80 degrees, between the acoustic beam and blood flow.

**Figure 2:**
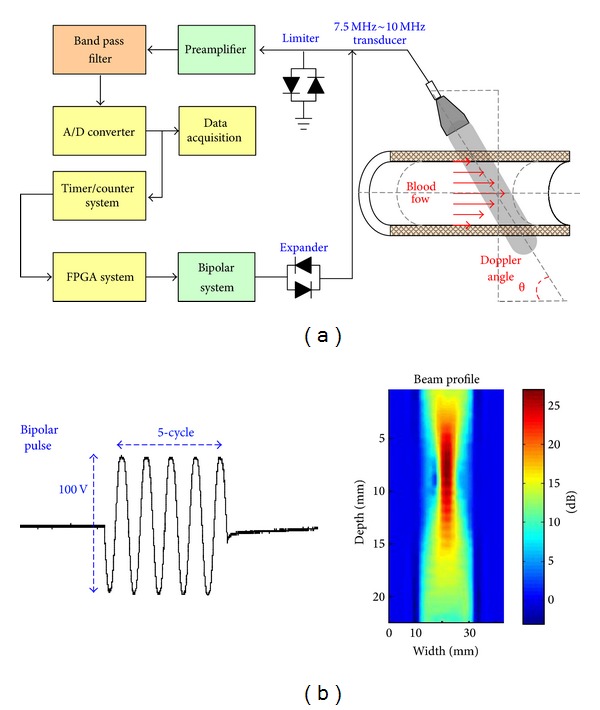
(a) The diagram of the proposed ultrasound device, (b) a 5-cycle, 7.5 MHz bipolar pulse with amplitude 100 Vpp.

In a narrowed access, turbulence occurs when blood flows through a stenosis cross-sectional area, increasing blood viscosity and flow resistance. In previous studies [27], Doppler shift usually encounters a velocity range of 0.10 m/sec~1.40 m/sec in clinical measurement and represents a frequency shift depending on the transducer frequency. The single transducer was excited by a pulse repetition frequency (PRF) using bipolar pulses [25, 27], as shown in Figure 2. Then, a 12 bit high-resolution digitizer with 200 MHz sampling frequency was used to record backscattering and original signals by PRF trigger synchronize control with FPGA control (DE2-70, Altera, San Jose, CA, USA). It has good resolution within a focusing zone of 10.0 mm × 10.0 mm and attenuation from 25.0 dB to 20.0 dB. Thus, the Doppler frequency shift can be calculated by those two signals in an embedded system. One advantage of the bipolar pulses design with FPGA control is that it drives a higher voltage and shorter pulse width than the traditional ultrasound system. Those features can provide better SNR of the signal and are suitable to detect blood changes in a hemodialysis access with the bipolar pulse control in the ranges of 7.5 MHz to 10 MHz. The bipolar pulse design is also a low-cost design that can be assembled easily into a handheld system.

### 2.2. Hemodynamic Analysis

A flow with periodic variations is a so-called pulsatile flow in small blood vessels or large arteries. In physical phenomena, the characteristics of the Doppler ultrasound velocity flow (DUFV) waveform can be used to analyze the pressure and propagation velocity of the physiologic conditions of the blood flow. According to the influence of dimensionless numbers, such as the Reynolds (Re) number, Womersley (*α*) number, and Strouhal (St) number, they can be quantified to describe the instability flow field, pulsatile flow, and oscillating flow mechanisms. They are defined as follows [28–31].

- Reynolds (Re) number is the ratio of inertial forces/viscous forces. Turbulent flow will occur at a high Reynolds numbers and is dominated by inertial forces, which tend to produce chaotic eddies, vortices, and instability flows.
- Womersley (*α*) number is the ratio of unsteady forces/viscous forces and is a dimensionless expression of the pulsatile flow frequency in relation to viscous effects. The vessel diameter decreases as the *α* number decreases in a stenotic cross-section.
- Strouhal (St) number is the ratio of oscillatory inertial forces/convective inertial forces and is a dimensionless number describing oscillating flow mechanisms. Its value also represents the dimensionless stroke volume.

The resistive (Res) index is also a parameter of pulsatile blood flow reflecting both vascular compliance (change in flow volume with a change in pressure) and vascular resistance in in vivo or in vitro studies. The Res index is defined as [26]

(2) Res=Vp−VmVp,

where *V*_*p*_ is the peak-systolic velocity (PSV) and *V*_*m*_ is the peak-diastolic velocity (PDV) through the Doppler ultrasound measurements. In the literatures [32], the cylindrical models with different diameters, viscosity, stroke displacement, and frequency were used to analyze flow instabilities. The critical peak Reynolds (Re_peak_) number was derived at a power law to indicate the flow instabilities for in vitro studies. It is calculated based on both the Womersley (*α*) numbers and Strouhal (St) numbers and indicates the transition flow to turbulence flow, which can be defined as [30]

(3) $$\begin{array}{c} \text{R}{\text{e}}_{\text{peak}}=169\alpha^{0.83}\text{S}{\text{t}}^{-0.27}. \end{array}$$

The supracritical Reynolds number, *Re*_supra_ = |*Re* − *Re*_peak_|, is correlated with body weight, female/male, vessel diameter, pulsatility index, and cardiac output. Therefore, *Re*_supra_ dimensionless can be used to define critical values along the cross-sectional AVS access in an in vivo study. In addition, the cross-section for every analysis plane is the noncircular duct in a blood vessel. The height and width are comparable, and the dimension for internal flow is taken to be the hydraulic diameter, *D*_*H*_, which can be considered as [31]

(4) $$\begin{array}{c} D_{H}=\frac{4A}{P},\;D_{H}=D\;\text{for}\;\text{a}\;\text{circular}\;\text{duct}\\ P=\overset{N_{l}}{\underset{l=0}{\sum }}L_{l},\;l=1,2,3,\ldots ,N_{l}, \end{array}$$

where *P* is the wetted perimeter, *L*_*l*_ is the length of each surface in contact with the aqueous body, and the spline interpolation is used for noncircular surface interpolation with *N*_*l*_ fractions. In noncircular ducts, the hydraulic diameter, *D*_*H*_, needs to be substituted for the diameter of a circular duct in the Reynolds number, Womersley number, and Strouhal number.

### 2.3. Experimental Setup

We recruited patients who consented to undergo follow-up examinations. This study was approved by the Institutional Review Board (IRB) of Kaohsiung Veterans General Hospital, Tainan Branch, under contract number VGHKS13-CT12-11. A total of 30 patients were enrolled. The participants comprised 9 females and 21 males aged between 42 and 89, with a mean age of 69.4 ± 13.13 years, and all participants had an AVF. The participants' mean dialysis duration was 1~16 years. Arteriovenous shunt (AVS) is a pathological physiology created on a patient's forearm and upper arm to facilitate hemodialysis treatment. Due to repeated puncturing of the AVS accesses and long-term use, the interior of the accesses can exhibit pathologic changes, resulting in the formation of a thrombus, intimal hyperplasia, and changes in the aneurysmal deformability of the access.

Maintenance of proper function is the most important issue for hemodialysis patients. Therefore, it is necessary to evaluate AVS functions in routine screening by confirming the specific degrees of stenosis (DOS) from X-ray images, angiographic images, and ultrasound images. In clinical research, vessel stenosis is defined as DOS > 0.50 reduction in luminal diameter judged by comparison with either the adjacent vessel or graft and can be defined as [7, 10]

(5) $$\begin{array}{c} \text{DOS}=1-{\left(\frac{d_{H}}{D_{H}}\right)}^{2}, \end{array}$$

where *D*_*H*_ is the hydraulic diameter of the normal graft or vessel in the direction of the blood flow and *d*_*H*_ is the hydraulic diameter of the stenosis lesion.

Preliminary screening results by physicians confirmed the specific degrees, including Class I: DOS < 0.30, Class II: 0.30 < DOS < 0.50, and Class III: DOS > 0.50, where DOS = 1.00 is total occlusion. Then, the proposed Doppler ultrasound device was carried out to measure the multisites' blood velocity as following the direction of blood flow from an arterial anastomosis site (A site) to a venous anastomosis site (V site), as shown in Figure 1.

## 3. Adaptive Color Relation Classifier

### 3.1. Quantitative Analysis with the Dimensionless Numbers

Multisite measurements were used to detect the blood velocity. Two dimensionless numbers were calculated and used to quantify the DOS, which can be expressed as follows.

*(i) Ratios of Re*_*supra*_. Divide an AVS access into two segments and suppose the same volume of blood through those; the ratios of *Re*_supra_ at the A site, loop site (L site), and V site are defined as

(6) Ratio(A)=|Re(A)−Resupra(A)Re(L)−Resupra(L)|=Resupra(A)Resupra(L),Ratio(L)=Resupra(L)Resupra(L)=1,Ratio(V)=|Re(V)−Resupra(V)Resupra(L)|=Resupra(A)Resupra(L),

where each site's quantity is the value of the quantity in the loop site, the so-called the value of per unit. Thus, the one advantage can be checked rapidly for gross changes at each measurement site. Another advantage of quantities is that they can be correlated with body weight, female/male, vessel diameter, and hemodynamic factors for any patient, AVF/AVG, and cross-sectional analysis.

*(ii) Resistive (Res) Index*. This index is an indicator of vessel resistance, its value increasing as the end-diastolic velocity decreases. The normal range for the common vessel waveform is shown in 0.50~0.65. Higher values indicate disease or stenosis [26, 27].

**Figure 3:**
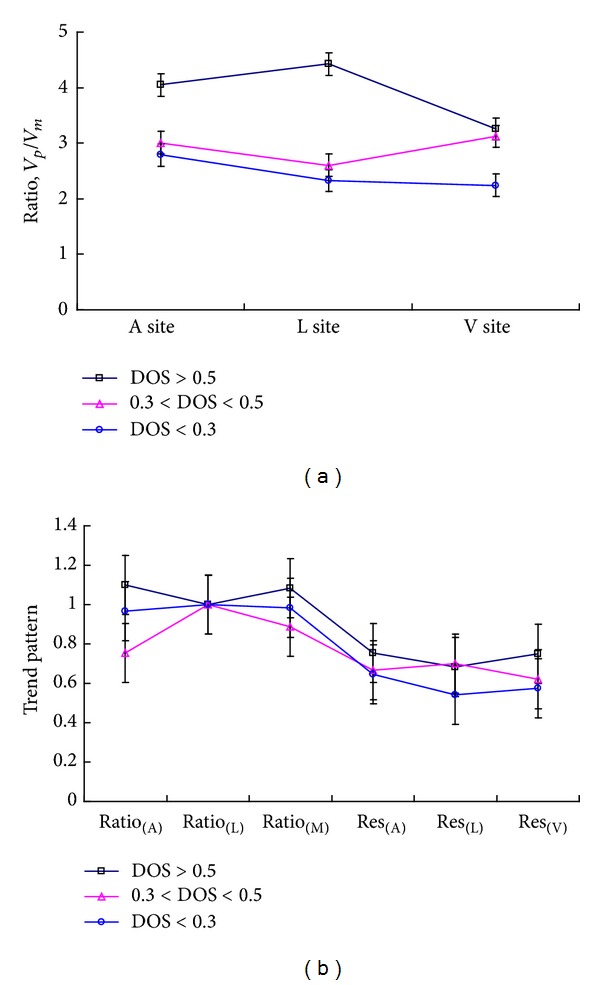
(a) The trends of ratios, *V*_*p*_/*V*_*m*_, (b) the trend patterns of six dimensionless numbers.

For 30 subjects, Table 1 shows the results of velocity measurements at the arterial anastomosis site (A), loop site (L), and venous anastomosis site (V), respectively. Because the AVS is a pulsatile access, the blood flow will mix high-velocity blood passing the lesion vessel and low-velocity blood downstream from the lesion vessel. The ratios of Re_supra_ and resistive (Res) indexes can be calculated with the key variables, *V*_*p*_ (PSV), *V*_*m*_ (PDV), hear rate, and hydraulic diameter. The Res indexes increase as the ratios of the *V*_*p*_/*V*_*m*_ (>3.0) increase at each measurement site, as shown in Figures 3(a) and 3(b). The ratios of Re_supra_ indicate the values approach to 1 for the same Re_supra_ through an AVS access, as the values gradually decrease for the degree, 0.3 < DOS < 0.5, and for values greater than 1 for the degree DOS > 0.5, as shown in Figure 3(b). The first three ratios are used to identify the laminar flow and flow instabilities from inflow to outflow, and the last three indexes are used to identify the intravascular resistances. Six parameters have specific ranges and can combine trend patterns to separate three degrees. According to these trend patterns of 30 subjects, we can systematically create training data for the adaptive color relation classifier.

### 3.2. Adaptive Color Relation Classifier Training

**Figure 4:**
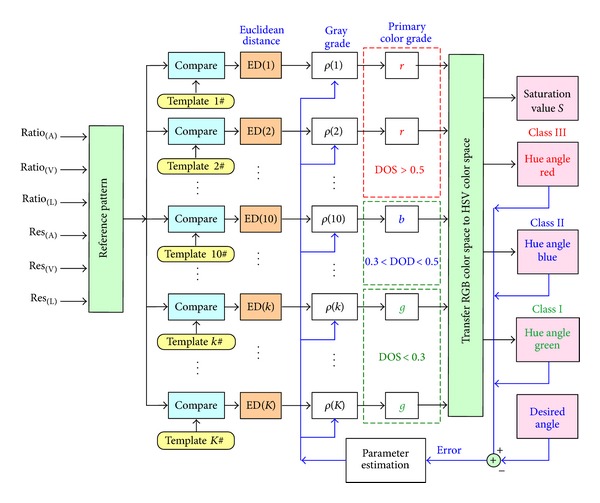
Structure of the adaptive color relation classifier.

Classification is the task of recognizing patterns into a few classes or categories. Based on similarity and dissimilarity, a mechanism with a learning algorithm can be designed to recognize patterns or features in diagnosis or screening applications [18]. In this study, a color relation analysis (CRA) method is proposed to develop a classifier, which possesses a flexible pattern mechanism with add-in and delete-off training data, and it does not require strict statistical methods or inference rules, as shown in Figure 4. Assuming Φ_*r*_ = [Ratio_(A)_(0), Ratio_(L)_(0), Ratio_(V)_(0), Res_(A)_(0), Res_(L)_(0), Res_(V)_(0)] = [*ϕ*_1_(0), *ϕ*_2_(0), *ϕ*_3_(0), *ϕ*_4_(0), *ϕ*_5_(0), *ϕ*_6_(0)] is a reference pattern, where *i* = 1, 2, 3,…, 6  (*n* = 6), and comparative patterns, Φ_*c*_(*k*) = [*ϕ*_1_(*k*), *ϕ*_2_(*k*), *ϕ*_3_(*k*),*ϕ*_4_(*k*), *ϕ*_5_(*k*), *ϕ*_6_(*k*)] are comparative patterns, where *k* = 1, 2, 3, …, *K*, as described in training data and represented below [18]

(7) $$\begin{array}{c} \left[\begin{array}{c} \Phi_{c}\left(1\right)\\ \Phi_{c}\left(2\right)\\ \vdots \\ \Phi_{c}\left(k\right)\\ \vdots \\ \Phi_{c}\left(K\right) \end{array}\right]=\left[\begin{array}{cccccc} \phi_{1}\left(1\right) & \phi_{2}\left(1\right) & \cdots & \phi_{i}\left(1\right) & \cdots & \phi_{6}\left(1\right)\\ \phi_{1}\left(2\right) & \phi_{2}\left(2\right) & \cdots & \phi_{i}\left(2\right) & \cdots & \phi_{6}\left(2\right)\\ \vdots & \vdots & \ddots & \vdots & \ddots & \vdots \\ \phi_{1}\left(k\right) & \phi_{2}\left(k\right) & \cdots & \phi_{i}\left(k\right) & \cdots & \phi_{6}\left(k\right)\\ \vdots & \vdots & \ddots & \vdots & \ddots & \vdots \\ \phi_{1}\left(K\right) & \phi_{2}\left(K\right) & \cdots & \phi_{i}\left(K\right) & \cdots & \phi_{6}\left(K\right) \end{array}\right], \end{array}$$

where *K* is the number of the comparative pattern. Compared with the reference pattern Φ_*r*_ and *k*th comparative pattern Φ_*c*_(*k*), the Euclidean distance, ED(*k*), can be expressed as a similarity degree between reference pattern, Φ_*r*_, and comparative pattern, Φ_*c*_(*k*), as

(8) $$\begin{array}{c} \text{ED}\left(k\right)=\sqrt{\overset{6}{\underset{i=1}{\sum }}{\left(\Delta \phi_{i}\left(k\right)\right)}^{2},}\;\Delta \phi_{i}\left(k\right)=\left|\phi_{i}\left(0\right)-\phi_{i}\left(k\right)\right|. \end{array}$$

If pattern, Φ_*r*_, is similar to any pattern, Φ_*c*_(*k*), the ED(*k*) will be a small value, and ED_min⁡_ = min⁡{ED(*k*)} ≤ ED(*k*) ≤ ED_max⁡_ = max⁡{ED(*k*)}. These indices can be used for pattern relation analysis. The overall indices ED(*k*) are converted to gray grade *ρ*(*k*) by nonlinear transformation, as [18]

(9) $$\begin{array}{c} \rho \left(k\right)=\xi \exp\left[-\xi \text{ED}\left(k\right)\right], \end{array}$$

where *ξ* is the recognition coefficient with interval (0, *∞*). Intensity adjustment is used to enhance the contrast by mapping the original intensity value to a new specific range. For AVS stenosis evaluation, these average gray grades can be stratified as three groups: Class I (normal), Class II, and Class III, represented as

(10) ρaveI=∑t=1NIρI(t)NI,ρaveII=∑t=1NIIρII(t)NII,ρaveIII=∑t=1NIIIρIII(t)NIII,

where *N*_I_, *N*_II_, *N*_III_, are the number of the respective groups. Minimum and maximum average grades are obtained as follows:

(11) ρmim=min⁡[ρaveI,ρaveII,ρaveIII],ρmax⁡=max⁡[ρaveI,ρaveII,ρaveIII],

where *ρ*_min⁡_ ≠ *ρ*_max⁡_. According to the hue-saturation-value (HSV) color model [18, 19], CRA is a mathematical model by transformations between the RGB primary color space and the HSV color space. Referring to the hue angle *H* ∈ [0, 360] as

(12) $$\begin{array}{c} H=\{\begin{array}{cc} \left[60^{{}^{\circ}}\times \left(\frac{g-b}{\Delta \rho }\right)+360^{{}^{\circ}}\right]\mathrm{mod}360^{{}^{\circ}}, & \text{if}\;\rho_{\max}=\rho_{\text{ave}}^{\text{III}},\\ 60^{{}^{\circ}}\times \left(\frac{b-r}{\Delta \rho }\right)+120^{{}^{\circ}}, & \text{if}\;\rho_{\max}=\rho_{\text{ave}}^{\text{I}},\\ 60^{{}^{\circ}}\times \left(\frac{r-g}{\Delta \rho }\right)+240^{{}^{\circ}}, & \text{if}\;\rho_{\max}=\rho_{\text{ave}}^{\text{II}}, \end{array} \end{array}$$

where Δ*ρ* = *ρ*_max⁡_ − *ρ*_min⁡_, and primary color grades, *r* (red), *g* (green), and *b* (blue) are, respectively, formulated as

(13) r=ρmax⁡−ρaveIIIΔρ,g=ρmax⁡−ρaveIΔρ,b=ρmax⁡−ρaveIIΔρ.

The saturation *S* and value *γ* are defined as

(14) $$\begin{array}{c} S=\frac{\gamma -\rho_{\min}}{\gamma },\;\rho_{\min}\neq \rho_{\max},\\ \gamma =\rho_{\max},\;\rho_{\max}\neq 0. \end{array}$$

The value of *H* is generally normalized to lie between 0° and 360°, where the centers of color distribution are 120°, 240°, and 360° for three degrees, respectively, and hue, *H*, has no geometric meaning when *ρ*_min⁡_ = *ρ*_max⁡_ and saturation, *S*, is zero. The parameter, *H*_*C*_, is utilized to identify the three classes as

(15) $$\begin{array}{c} H_{C}=\left[\frac{H}{360^{{}^{\circ}}}\right],\;H_{C}\in \left[0,1\right], \end{array}$$

where the critical decision *H*_*C*_ = [Class I, Class II, Class III] = [2/3, 1/3, 1]. The concept of the proposed CRA is derived from the HSV color model to describe perceptual color relationships for AVS stenosis estimation. The saturation, *S*, varies from 0.0 to 1.0, and the corresponding colors vary from unsaturated (value 0) to fully saturated (value 1). In this study, parameter, *H*_*C*_, is used to identify the possible degree; however, the choice of recognition coefficient, *ξ*, is a limitation. As its value gradually increased, *ξ* ≫ 1, gray grades were distinctly separated into three degrees, and the decision-making might become increasingly nonlinear for a high-dimensional classification space, which will affect the screening accuracy.

Therefore, the CRA classifier with an optimal recognition coefficient, *ξ*, can enhance the accuracy. Consider the mean squared error function (MSEF); the objective is intended to minimize the MSEF, as

(16) $$\begin{array}{c} \min\left(\text{MSEF}\right)=\min\left(\frac{1}{K}\overset{K}{\underset{k=1}{\sum }}{\left[T\left(k\right)-H_{C}\left(k\right)\right]}^{2}\right)\leq \varepsilon , \end{array}$$

where *T*(*k*) is the desired degree for the *k*th training data. However, *H*_*C*_ is a nonlinear function and its partial differential equation is difficult to obtain using the conventional gradient method.

This study proposes the adaptive color relation classifier with adaptability to adjust the recognition coefficient to enhance screening accuracy with add-in and delete-off training data. For an adaptive classifier design, the particle swarm optimization (PSO) algorithm is an evolutionary method to solve optimization problems for time-varying/dynamic search spaces. The property of a PSO algorithm searches using multiple particles that modify their search positions around a multidimensional search space, until the unchanged positions have been achieved. Each position is adjusted based on the past action experiences and the dynamically altering the velocity of each particle [20, 21]. Let *ξ*_*gp*_ be the current position of the *g*th agent at iteration number *p*, agent *g* = 1, 2, 3, …, *G*, where *G* is the population size. Multiple particles form a population and are represented by a *G*-dimensional vector, as *ξ* ^*p*^ = [*ξ*_1_^*p*^, *ξ*_2_^*p*^, …, *ξ*_*g*_^*p*^, …, *ξ*_*G*_^*p*^]. The modification of position, *ξ*_*g*_^*p*^, can be represented by velocity, Δ*ξ*_*g*_^*p*^, Δ*ξ*_*g*_^*p*^ = [Δ*ξ*_1_^*p*^, Δ*ξ*_2_^*p*^, …, Δ*ξ*_*g*_^*p*^, …, Δ*ξ*_*G*_^*p*^]. The mathematical representation is given by [20] the following.

Velocity:

(17) Δξgp+1=ωΔξgp+c1rand1(ξbestg−ξgp)    +c2rand2(ξbest−ξgp),c1=(b1−a1)ppmax⁡+a1, c2=(b2−a2)ppmax⁡+a2.

**Figure 5:**
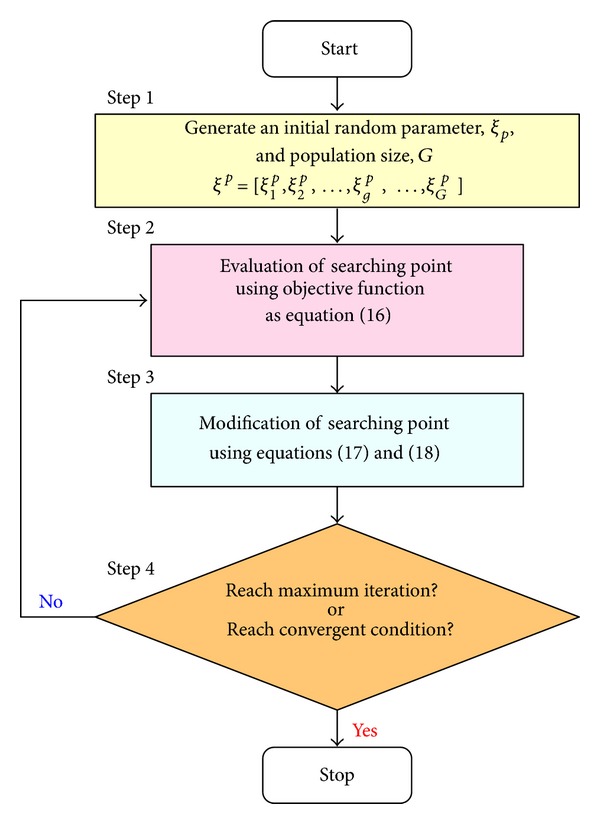
The flow chart of PSO algorithm.

Position:

(18) $$\begin{array}{c} \xi_{g}^{p+1}=\xi_{g}^{p}+\Delta \xi_{g}^{p+1}, \end{array}$$

where *ξ*_best_ is the global best in the group, *ξ*_best_*g*__ is the individual best, *ω* is the inertia weight, rand_1_ and rand_2_ are the uniformly random numbers between 0 and 1, and *c*_1_ and *c*_2_ are the acceleration parameters, where the first term is the “cognitive component” and the second term is the “social component.” Parameters, *a*_1_, *b*_1_, *a*_2_, and *b*_2_, are constant values, of which the experienced values are *c*_1_ from 2.5 to 0.5 and *c*_2_ from 0.5 to 2.5, respectively [21], and *p*_max⁡_ is the maximum number of allowable iterations. Therefore, a PSO algorithm with time-varying acceleration coefficients (TVAC) [20] could efficiently converge to the global optimal solution. The flow chart of the PSO algorithm is shown in Figure 5.

## 4. Experimental Results and Discussion

**Figure 6:**
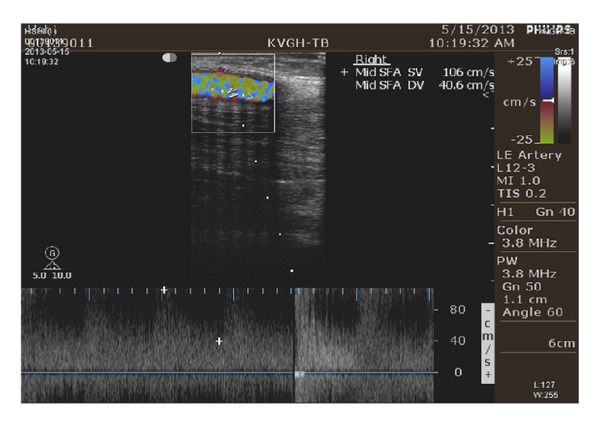
Ultrasonic image examination date: May 15, 2013, Kaohsiung Veterans General Hospital, Tainan Branch.

Following the direction of blood flow from the arterial anastomosis site (A) to the venous anastomosis site (V), multisite measurements were used to detect the blood velocities using the Doppler ultrasound device. A 7.50 MHz transducer was excited by sinusoidal tone bursts with 5-cycle long pulses at pulse repetition frequencies (PRFs) of 19.48 kHz~27.27 kHz. For the pulsatile flow experiments, the stroke rate was set at 7.5~30 beats/min at a peak flow velocity of 1.00 m/sec~1.40 m/sec, using a PRF trigger with FPGA control [22, 23]. The relevant hemodynamic parameters and transducer parameters for the experimental setup are shown in Table 2. A 12 bit high-resolution digitizer with 200 MHz sampling frequency was used to record backscattering and original signals by PRF trigger synchronize control. Then one can calculate the Doppler frequency shift by those two signals in the PC system. The velocities, ratios of superacritical Reynolds (*Re*_supra_) numbers, and resistive (Res) indexes were calculated using (2) and (3). The proposed adaptive CRA based classifier was developed on a PC AMD Athlon II ×2 245 2.91 GHz with 1.75 GB RAM and Matlab software (Mathworks, Natick, MA). To demonstrate the effectiveness of the proposed method for AVS stenosis evaluation, 40 subjects were chosen (IRB: VGHKS13-CT12-11) to be divided into 30 training data and 10 testing data. Preliminary diagnosis results confirmed the specific degree by ultrasonic image examination and observation by clinical physicians, as shown in Figure 6. Feasibility tests and case studies were used to validate the proposed estimation model, as detailed below.

### 4.1. Feasibility Tests with the Adaptive CRA

**Figure 7:**
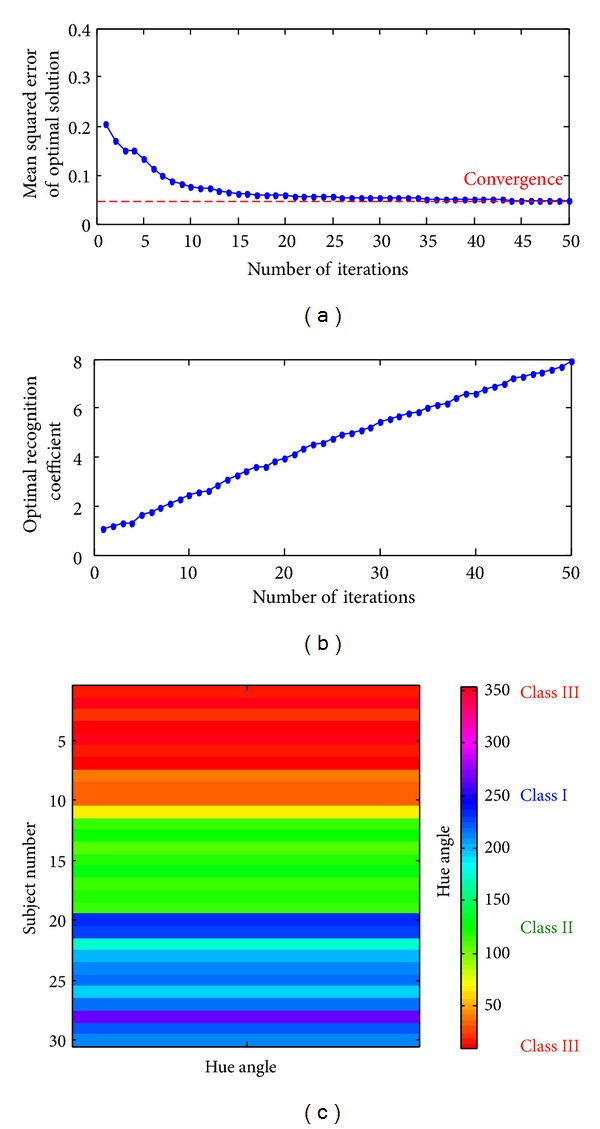
(a) The mean squared error of optimal solution versus the number of iteration training, (b) the optimal recognition coefficient versus the number of iteration training, and (c) the training results with the adaptive color relation classifier.

The 30 subjects were divided into three groups as training data. The overall training results are shown in Figure 7. The performance of the CRA classifier is affected by the nonlinear gray grade transformation as seen in (9). As the recognition coefficient, *ξ*, gradually increased, gray grades were distinctly separated into three groups. The decision might become increasingly nonlinear for a high-dimensional classification space. Therefore, recognition coefficient, *ξ* ≫ 0, was selected, and the hue angle, *H*_*C*_, had high confidence to transform between the RGB primary color space and the HSV color space. The saturation value, *S*, also indicated the confidence indices and confirmed the values approached to 1.

According to the training data, the CRA classifier has 6 inputs and 3 outputs, as shown in Figure 4. The first stage is calculation of the Euclidean distances (EDs) between reference patterns and comparative patterns. The most similar choice is the smallest ED. Then the overall EDs are converted to gray grades and are grouped into three classes. Then, the hue angle, *H*_*C*_, is carried out to identify the possible class. In the second stage, updating the recognition coefficient is performed by the proposed PSO algorithm. For the adaptive application, the PSO with time-varying acceleration coefficients (TVAC) is given by population size, *G* = 20, for each iteration, acceleration coefficients, *a*_1_ = 2.5 and *b*_1_ = 0.5, for the cognitive component, acceleration coefficients, *a*_2_ = 0.5 and *b*_2_ = 2.5, for the social component [20, 21], and the maximum allowable number, *p*_max⁡_ = 100. With the convergent condition, MESF < *ε* = 0.05, it can be seen that the adaptive CRA can find the optimal parameter, *ξ*_best_ = 7.9883, and minimize the mean squared error, as shown in Figures 7(a) and 7(b). The training results with the adaptive color relation classifier are shown in Figure 7(c).

In comparison, Table 3 shows comparative performances using the proposed method and multilayer networks, such as artificial neural networks (ANNs) and support vector machines (SVMs) [4, 5, 8]. Stethoscope auscultation was used to measure the phonoangiography (PCG) signals. Then feature selection takes the specific frequencies and the magnitude of the characteristic frequencies using the time-frequency methods and Burg methods [6, 7, 9, 10]. It provides time-frequency resolution to extract features from nonstationary PCG signals. However, significant frequencies need trial and error procedures using the cascaded low-pass/high-pass filters and down-sampling/up-sampling operations. Its technique is limited by the preprocessing time, computational time, and significant feature selection. In addition, multilayer network classifier is a mechanism that has been widely applied in the continuous modeling system with iteration training, such as tuning network parameters and automatic target adjustment. Briefly, the learning performance heavily relies on the choices of the initial conditions, learning rates, and convergent conditions. The training process and classification efficiency become a major problem when handling huge training data, and it also becomes time consuming, without a guaranteed global minimum.

In contrast, the PSO algorithm is an optimization technique that avoids the drawbacks of the least mean square algorithm and gradient descent method. Due to difficulty in obtaining the partial derivatives of the nonlinear mean squared error function as (16), swarm intelligence can guide searches using multiple particles rather than individuals. Individuals in a swarm approach the optimum through the present solution, previous experiences, and other individuals' experiences and can avoid trapping at a local minimum. An optimization solution is sensitive to certain control factors, such as the population sizes, time-varying acceleration coefficients, and the number of iteration training [33]. However, we have suggested that the suitable control factors terminate the PSO algorithm to find the optimum solutions. Using 30 subjects, our comparison indicates CRA using PSO algorithm has higher accuracies, 95~100%, than multilayer networks with accuracies of 70~90%.

### 4.2. Physical Examinations

Using testing data from the other 10 subjects who agreed to participate in this study, the overall testing results are shown in Table 4. The tests used at least 10 records at the measurement sites to calculate the flow velocities and dimensionless numbers. We have three main degrees to decide the occlusion levels, and the hue angles, *H*, correspond to define the level to quantify the similarity in each class. The hue angles can determine the similarity levels, and more likely approach to the around of angles, 240° (blue), 120° (green), and 0°/360° (red) from Class I to Class III, respectively. If *H* is very similar, its value will be close to the primary color grades. From these physical examinations, we observed the dysfunction of AVS could be screened by the proposed classifier.

The CRA based classifier used a straightforward mathematical operation to construct a pattern recognition model with expandable or reducible training data. Euclidean distances are used to express the similarity degree between a reference pattern and comparative patterns which represents the largest similarity. It was also designed as an adaptive pattern mechanism with an adjustable recognition coefficient to enhance the higher screening accuracies. Thus, it can reduce the training data requirements.

## 5. Conclusion

A CRA based classifier was proposed to screen the degrees of stenosis for an arteriovenous access occlusion evaluation. Doppler ultrasound is a noninvasive measurement support to measure blood flow at multiple sites in an in vivo examination. The Doppler acoustic device with the suggested 7.5 MHz~10.0 MHz central operation frequency and the sinusoidal tone bursts provides a good resolution that allows the measurement of the blood velocities in the arteriovenous access. The dimensionless numbers, the ratios of supracritical Reynolds and resistive indexes, can be calculated with the peak-systolic velocities, the peak-diastolic velocities, and the heart rates. The trends in the ratios of the supracritical Reynolds numbers and the resistive indices indicate a promising way to evaluate the occlusion levels at multiple measurement sites. The proposed classifier has a flexible pattern mechanism with add-in and delete-off training data and the capacity to adjust the recognition coefficient to enhance accuracy. For 40 subjects divided into training and testing groups, the proposed classifier was validated to survey the level of occlusion for the clinician. The results can be presented in colors as red, green, and blue and can be implemented in computer graphics applications. Further study could also implement the proposed screening method in an advanced embedded system or a programmable microprocessor and could be used to allow integration with handheld healthcare systems.

## Figures and Tables

**Table 1:** The results of velocity measurements.

DOS/average velocity	Measurement site		
	Arterial anastomosis site (A)	Loop site (L)	Venous anastomosis site (V)
<0.30 (12)			
*V*_*p*_(cm/sec)	103.20 ± 18.35	64.60 ± 28.05	82.56 ± 25.30
*V*_*m*_ (cm/sec)	37.00 ± 10.80	28.83 ± 16.61	35.43 ± 17.37
Δ*P* (mmHg)	4.26 ± 0.13	1.67 ± 0.31	2.72 ± 0.26
0.30~0.50 (11)			
*V*_*p*_ (cm/sec)	106.70 ± 28.80	54.91 ± 21.54	77.79 ± 18.54
*V*_*m*_ (cm/sec)	35.49 ± 19.96	16.61 ± 8.69	29.87 ± 15.81
Δ*P* (mmHg)	4.55 ± 0.33	1.21 ± 0.19	2.42 ± 0.14
>0.50 (7)			
*V*_*p*_ (cm/sec)	72.21 ± 40.58	25.61 ± 9.62	66.03 ± 42.12
*V*_*m*_ (cm/sec)	17.84 ± 8.7	7.86 ± 3.84	14.92 ± 14.19
Δ*P* (mmHg)	2.09 ± 0.66	0.26 ± 0.04	1.74 ± 0.71

Note: the pressure drop, Δ*P* (mmHg), can be related to the velocity, *V*_*p*_ (m/sec), and is defined by Δ*P* = 4(*V*_*p*_)^2^ [26].

**Table 2:** The related parameters of experimental setup.

Hemodynamic parameters	
Blood density	1055 kg/m^3^
Hematocrit (Ht)	40% for a normal adult
	*μ*_plasma_ = 0.0035 pas
Dynamic viscosity	0.01063 Ns/m^2^
	Room temperature = 26°C
Heart rate	1.00~1.25 Hz
Hydraulic diameter	Ultrasound image examination for Follow-up examination

Specification of transducer	
Center operation frequency, *f*_0_	7.50 MHz
Output voltage	±50 V (*V*_pp_ = 100 V)
Pulse repetition frequency, PRF	19.48 kHz~27.27 kHz
	Velocity range: 1.0 m/s~1.4 m/s
Transmitted pulse duration	0.66 *μ*s
Pulse cycle	5 cycles
Doppler angle	45~60 degrees

**Table 3:** Comparison of performances between the proposed method and artificial neural network (ANN).

Task	Method	
	CRA	ANN and SVM
Network structure	Hybrid decision-making and network structure	Multilayer network

Training data	Yes	Yes
	Minor	Major

Feature selection	Hemodynamic analysis with dimensionless numbers	Time-frequency analysis and Burg method [6, 7, 9, 10]

Inference/learning algorithm	PSO algorithm [20, 21]	(i) Least mean square algorithm
		(ii) Gradient descent method

Parameter assignment	Yes	Yes
	Minor	Major

Adjustable parameter	Yes	Yes
	Minor	Major

Iteration training	Yes	Yes

Convergent condition	Yes	Yes

**Table 4:** Results of physical examinations.

		Measurement site						
Number/DOS		A		L		V		Hue angle, *H*/saturation, *S *
		Ratio (A)	Res	Ratio (L)	Res	Ratio (V)	Res	
1	0.9129	1.11	0.733	1.00	0.71	1.02	0.63	11.6479
								0.6480
2	0.9056	1.41	0.87	1.00	0.50	1.29	0.74	12.2690
								0.9707
3	0.5346	1.21	0.66	1.00	0.60	1.20	0.65	21.9924
								0.9023

4	0.4866	0.70	0.72	1.00	0.59	0.98	0.67	58.0319
								0.6140
5	0.4066	0.89	0.78	1.00	0.74	0.89	0.66	134.8687
								0.5659
6	0.3960	0.88	0.88	1.00	0.76	0.75	0.61	120.8012
								0.7880

7	0.1845	0.85	0.66	1.00	0.60	0.98	0.63	198.2063
								0.6034
8	0.1830	0.93	0.65	1.00	0.72	0.82	0.53	209.9895
								0.5357
9	0.1467	0.97	0.64	1.00	0.51	1.02	0.66	191.1797
								0.8221
10	0.0985	1.04	0.63	1.00	0.44	0.85	0.37	218.0953
								0.9072
